# HMB Improves Lipid Metabolism of Bama Xiang Mini-Pigs via Modulating the *Bacteroidetes*-Acetic Acid-AMPKα Axis

**DOI:** 10.3389/fmicb.2021.736997

**Published:** 2021-08-16

**Authors:** Jie Zheng, Changbing Zheng, Bo Song, Qiuping Guo, Yinzhao Zhong, Shiyu Zhang, Lingyu Zhang, Geyan Duan, Fengna Li, Yehui Duan

**Affiliations:** ^1^CAS Key Laboratory of Agro-Ecological Processes in Subtropical Region, Hunan Provincial Key Laboratory of Animal Nutritional Physiology and Metabolic Process, National Engineering Laboratory for Pollution Control and Waste Utilization in Livestock and Poultry Production, Institute of Subtropical Agriculture, Chinese Academy of Sciences, Changsha, China; ^2^College of Advanced Agricultural Sciences, University of Chinese Academy of Sciences, Beijing, China

**Keywords:** β-hydroxy-β-methylbutyrate, lipid metabolism, gut microbiota, acetic acid, AMPKα, Bama Xiang mini-pigs

## Abstract

Here, we used Bama Xiang mini-pigs to explore the effects of different dietary β-hydroxy-β-methylbutyrate (HMB) levels (0, 0.13, 0.64 or 1.28%) on lipid metabolism of adipose tissue. Results showed that HMB decreased the fat percentage of pigs (linearly, *P* < 0.05), and the lowest value was observed in the 0.13% HMB group. Moreover, the colonic acetic acid concentration and the relative *Bacteroidetes* abundance were increased in response to HMB supplementation (*P* < 0.05). Correlation analysis identified a positive correlation between the relative *Bacteroidetes* abundance and acetic acid production, and a negative correlation between fat percentage and the relative *Bacteroidetes* abundance or acetic acid production. HMB also upregulated the phosphorylation (p) of AMPKα, Sirt1, and FoxO1, and downregulated the p-mTOR expression. Collectively, these findings indicate that reduced fat percentage in Bama Xiang mini-pigs could be induced by HMB supplementation and the mechanism might be associated with the *Bacteroidetes*-acetic acid-AMPKα axis.

## Introduction

Obesity has increased at an alarming rate over the past years, subsequently resulting in diabetes and other metabolic diseases (Duan Y.H. et al., 2017; Duan Y. et al., 2017). There is a wealth of data indicating that an effective strategy to combat obesity is to reduce the weight of adipose tissue (Yao et al., 2016; Yin et al., 2018). Notably, there are regional differences in the sensitivity of adipose tissue depots in response to dietary manipulations and the most sensitive depot is the subcutaneous white adipose tissue (WAT) (Fickova et al., 1998; Gaiva et al., 2001; Rodríguez et al., 2002). Moreover, subcutaneous WAT accounts for ∼85% total fat mass, and a lower subcutaneous WAT is more protective compared to visceral WAT (Booth et al., 2018). Therefore, subcutaneous WAT was a focus of this study, with the goal to understand dietary nutrients to combat obesity. Evidence in the literature has demonstrated that WAT has a variety of well-documented functions, including serving as an energy storage site, regulating glucose and lipid homeostasis, and producing adipokines (Cummins et al., 2014). The two primary metabolic activities of WAT (lipogenesis and lipolysis) cooperate to maintain the relative constancy of body fat under normal conditions (Proença et al., 2014). In humans, excessive body fat contributes to obesity and diabetes (Zheng J. et al., 2021). In the meat industry, the increased WAT mass has a deleterious effect on production efficiency and meat quality (Lebret and Mourot, 1998). Therefore, maintaining an appropriate WAT mass is an important objective pursuit of animal production and human health.

Leucine functions as a direct-acting nutrient signal in WAT to affect lipid metabolism, thus favoring adiposity reduction (Yao et al., 2016; Zhang et al., 2020). Despite these positive outcomes, multiple lines of evidence have pointed out that elevated levels of circulating leucine may precede or coincide with insulin resistance and cardiovascular dysfunction, thus seriously threatening human health (Felig et al., 1969; Yang et al., 2015). In explanation of these observations, previous studies have hypothesized that the ability of adipocytes to degrade or oxidize leucine is lost (Gannon et al., 2018). Interestingly, β-hydroxy-β-methylbutyrate (HMB), a metabolite of leucine, has been reported to share similar regulatory effects on lipid metabolism with leucine (Duan et al., 2018). More importantly, HMB cannot be reversibly converted to leucine (Nissen and Abumrad, 1997). Of note, a growing number of evidence has demonstrated convincingly that dietary HMB supplementation is an effective way to regulate lipid metabolism. For example, HMB treatment (0.62%, 45 days) gave rise to fat loss in growing pigs (Duan et al., 2018). Other experiments conducted in rodent models also confirmed that animals having access to HMB showed a decline in fat mass (Wilson et al., 2012; Park et al., 2013). Thus, we shifted the focus from leucine to HMB, which may provide an alternative for safe and efficacious regulation of lipid metabolism.

Alterations in the gut-microbiota community have been reported to be associated with the occurrence and development of obesity (Marchesi et al., 2016; Wang Z. et al., 2018). The metabolic activity of the gut microbiota can be linked to host body energy homoeostasis by short-chain fatty acids (SCFAs), which are produced by gut microbiota catabolizing dietary fibers (Topping and Clifton, 2001). There is compelling evidence that nutrients can attenuate obesity by reprogramming gut microbiota (Xu et al., 2017; Yin et al., 2018; Zhang et al., 2019; Wang et al., 2020; Zhong et al., 2020). Using a rodent model, we also found that HMB could mitigate lipid metabolism disorders by reprogramming gut microbiota, as manifested by the increased ratio of *Bacteroidetes* to *Firmicutes* and elevated *Bacteroidetes*-produced propionic acid (Duan et al., 2019). However, the literature on the possible relationship between HMB treatment and gut microbiota is dominated by studies in rodents, whether the beneficial effects of HMB in larger mammals including pigs are mediated by gut microbiota requires further interrogation.

Bama Xiang mini-pig, a fat pig breed, is an optimal model to explore the regulation role of diets on obesity due to its anatomical and physiological similarities with humans (Chen et al., 2009; Jiang et al., 2015; Koopmans and Schuurman, 2015; Niu et al., 2017). Therefore, in this study, Bama Xiang mini-pigs were used to (1) investigate the effects of dietary HMB levels on lipid metabolism in the WAT, and (2) illustrate the potential mechanisms underlying the action of HMB on lipid metabolism. It was hypothesized that the optimal dietary HMB level could reduce fat accumulation in the WAT of Bama Xiang mini-pigs through regulating gut microbiota.

## Materials and Methods

### Animals and Diets

The experiments in the present study were approved by the Animal Welfare Committee of the Institute of Subtropical Agriculture, Chinese Academy of Sciences. The ethic approval number is ISA-2017-023.

Thirty-two Bama Xiang mini-pigs (8.58 ± 0.40 kg, barrow) were selected and randomly allotted to four dietary treatments (8 piglets per treatment). The HMB-Ca level of the four dietary treatments were as follows: 0 (control), 0.13, 0.64, and 1.28%. The HMB-Ca (purity ≥ 99.0%) was obtained from Jiangyin TSI Pharmaceutical Co., Ltd. All diets were isoenergetic and isonitrogenous (Zheng C. et al., 2021), and met the nutritional needs for growing-mini pigs (Gao and Liu, 2009). The pigs raised individually in cages with *ad libitum* access to diets and water. This experiment lasted for 60 days.

### Sample Collection and Fat Percentage Measurement

At the end of the experiment, pigs were fasted overnight (12 h) and then slaughtered by electrical stunning (250 V, 0.5 A, for 5–6 s) and exsanguinating. After slaughter, samples of WAT including DSA and ASA were rapidly excised from the right side of the carcass. The samples were then either stored at −20°C for the determination of fatty acid composition or quickly frozen in liquid nitrogen and then stored at −80°C for the analysis of Western blotting. Meanwhile, carcass weight and fat mass weight were recorded, and the fat percentage was calculated by dividing the fat mass weight by carcass weight. Subsequently, the colon was quickly separated and the colonic contents were collected from a region 10 cm posterior to the ileocecal valve into sterile tubes and stored at −80°C for further analysis.

### Determination of Fatty Acid Composition

The fatty acid composition of DSA and ASA was analyzed via gas-liquid chromatography of methyl esters using an Agilent 7890A GC as previously described (Yin et al., 2000; Liu et al., 2015). The following indices were calculated based on fatty acid composition: the sum of saturated fatty acids (SFA), monounsaturated fatty acids (MUFA), and polyunsaturated fatty acids (PUFA), respectively. Then, the ratio of PUFA to SFA and the ratio of n-6 to n-3 PUFA were assessed.

### Adipose Tissue Histologic Analysis

At the time of sacrifice, samples of DSA and ASA (1 cm^3^) were collected and fixed in 4% paraformaldehyde in PBS (pH 7.3) for paraffin sections and hematoxylin and eosin staining as previously described (Zhang et al., 2021). Tissue sections were imaged at 100 × magnification using a microscope (Eclise Ci-L, Nikon, Japan). The adipocyte size (10 fields/sample) was quantified using DIXI3000 (Leica Camera, Wetzlar, Germany).

### Determination of Colonic SCFAs Concentrations

About 1 g of colonic contents were used to measure the concentrations of SCFAs (acetate, propionate, butyrate, isobutyrate, valerate, and isovalerate) by using an Agilent 6890A gas chromatography (Agilent Technologies, Santa Clara, CA, United States) according to our previous study (Yin et al., 2018).

### Gut Microbiota Analysis

Gut microbiota in the colon of Bama Xiang mini-pigs were determined as previously described (Duan et al., 2019; Song et al., 2019). Alpha diversity indices, including Shannon, Simpson, Chao1, and ACE, were used to evaluate the diversity and richness of gut microbiota (Lemieux-Labonte et al., 2017).

### Western Blotting Analysis

The relative protein expression of phosphorylated AMPKα (p-AMPKα), FoxO1 (p-FoxO1), mTOR (p-mTOR), and Sirt1 (p-Sirt1) in the DSA and ASA were determined as previously described (Duan et al., 2013). All the primary antibodies were purchased from Cell Signaling Technology (Danvers, MA) and the secondary antibodies were obtained from Thermo Scientific Inc. (Waltham, MA, United States). The protein bands were visualized by a chemiluminescent reagent (Pierce, Rockford, United States) with a ChemiDoc XRS system (Bio-Rad, Philadelphia, PA, United States). We quantified the resultant signals using Alpha Imager 2200 software (Alpha Innotech Corporation, CA, United States) and normalized the data with the value of the inner control GAPDH.

### Statistical Analysis

Data were analyzed using one-way analysis of variance (ANOVA) using the SAS version 8.2 (SAS Institute Inc., Cary, NC, United States) software followed by Duncan’s multiple comparison test. Orthogonal polynomial contrasts were performed to determine linear and quadratic effects of increasing dietary HMB on the measured indicators using SPSS 22.0 software (SPPS Inc., Chicago, IL, United States). Results are presented as means ± standard errors. Differences between significant means were considered statistically different at *P* < 0.05. Probability values between 0.05 and 0.10 were considered trends.

## Results

### Fat Percentage and Adipocyte Size

**FIGURE 1:**
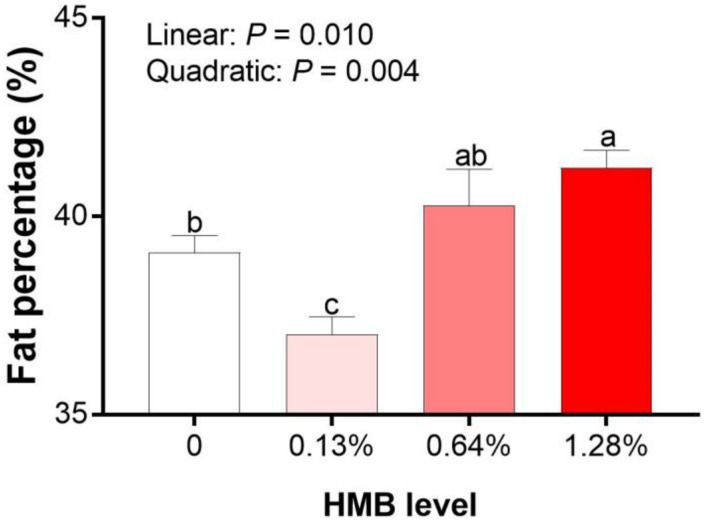
Effects of dietary HMB supplementation on fat percentage of Bama Xiang mini-pigs. Data are presented as mean ± SEM (*n* = 8). ^*a,b,c*^ Values with different letters are significantly different among dietary HMB treatments (*P* < 0.05).

As shown in Figure 1, linear effects of increased HMB levels on fat percentage was observed, with the highest value observed in the 1.28% HMB group and the lowest value observed in the 0.13% HMB group (*P* < 0.05).

**FIGURE 2:**
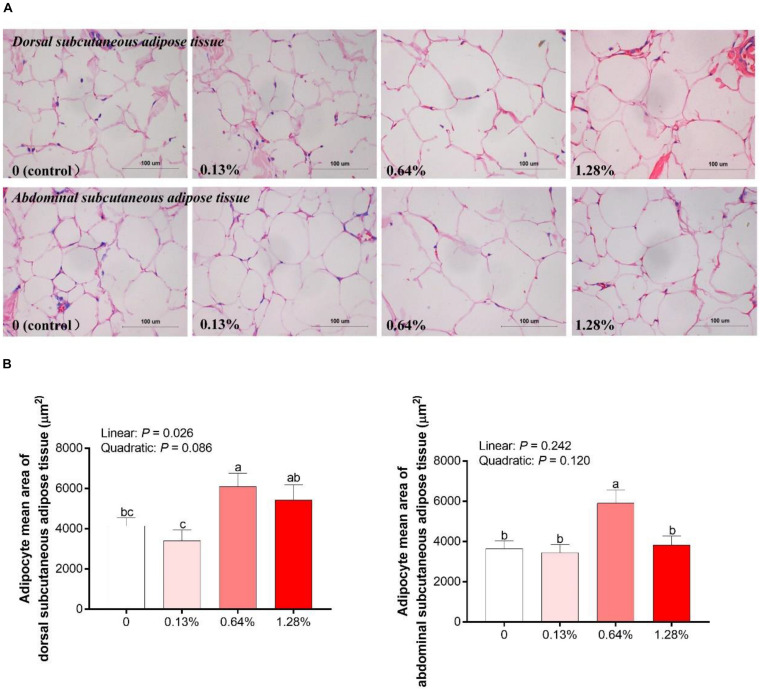
Analysis of hematoxylin and eosin slices of adipocytes in Bama Xiang mini-pigs offered four diets **(A)** and the adipocyte mean area of adipocytes **(B)** in the adipose tissue of Bama Xiang mini-pigs. Data are presented as mean ± SEM (*n* = 8). ^*a,b,c*^ Values with different letters are significantly different among dietary HMB treatments (*P* < 0.05).

As presented in Figure 2, HMB linearly increased the adipocyte mean area of DSA, and the highest/lowest value was obtained in the 0.64% HMB group/0.13% HMB group, respectively (*P* < 0.05). The adipocyte mean area of ASA in the 0.64% HMB group was higher than that in the other three groups (*P* < 0.05), and there was no significant difference among the three groups.

### Fatty Acid Composition

As depicted in Table 1, dietary HMB supplementation significantly influenced the fatty acid composition in the DSA. HMB supplementation linearly increased the concentration of C18:3 n3, with the highest value observed in the 0.13% HMB group (*P* < 0.05). As dietary HMB levels increased, the concentration of C18:2 n6c showed a downward trend (quadratic, *P* = 0.053). The concentration of C20:4 n6 was decreased in the HMB groups compared with the control (linear and quadratic, *P* < 0.05). The n6:n3 PUFA ratio was the highest in the 1.28% HMB group and the lowest in the 0.13% HMB group, with intermediate values observed in the other two groups (quadratic, *P* < 0.05). HMB quadratically decreased the sum of PUFA (*P* < 0.05) and the ratio of PUFA:SFA (*P* = 0.085). Dietary treatments did not significantly affect the sum of SFA and MUFA (*P* > 0.05).

**TABLE 1:** Fatty acid composition of dorsal subcutaneous adipose tissue in Bama Xiang mini-pigs fed the diets with various levels of HMB (% of total fatty acids).

Items	Dietary levels of HMB, %				SEM	*P*-value^1^		
	0	0.13	0.64	1.28		ANOVA	Linear	Quadratic
C10:0	0.10^*ab*^	0.09^*b*^	0.11^*ab*^	0.12^*a*^	0.041	0.074	0.034	0.059
C12:0	0.13	0.11	0.13	0.13	0.052	0.517	0.960	0.641
C14:0	1.76	1.56	1.73	1.80	0.165	0.299	0.488	0.302
C16:0	27.13	26.98	27.25	27.82	0.319	0.322	0.121	0.167
C16:1	1.57	1.42	1.62	1.65	0.176	0.412	0.338	0.455
C17:0	0.28^*a*^	0.25^*ab*^	0.22^*b*^	0.23^*b*^	0.065	0.048	0.012	0.022
C18:0	18.01	18.91	18.06	17.63	0.389	0.336	0.391	0.291
C18:1 n9t	0.12	0.13	0.12	0.12	0.054	0.747	0.714	0.600
C18:1 n9c	35.55	35.91	36.61	35.86	0.388	0.495	0.463	0.413
C18:2 n6c	11.05^*a*^	10.43^*ab*^	10.16^*b*^	10.82^*ab*^	0.285	0.113	0.464	0.053
C20:0	0.35^*ab*^	0.37^*a*^	0.36^*ab*^	0.31^*b*^	0.071	0.096	0.122	0.039
C20:1	1.89	1.94	1.89	1.71	0.203	0.641	0.322	0.424
C18:3 n3	0.52^*ab*^	0.56^*a*^	0.46^*b*^	0.46^*b*^	0.087	0.023	0.022	0.061
C20:2	0.93	0.88	0.82	0.82	0.124	0.389	0.087	0.223
C20:3 n6	0.15	0.14	0.13	0.14	0.049	0.424	0.163	0.301
C22:1 n9	0.20	0.18	0.17	0.17	0.057	0.196	0.046	0.100
C20:4 n6	0.24^*a*^	0.22^*b*^	0.21^*b*^	0.21^*b*^	0.048	0.014	0.003	0.006
SFA^2^	47.75	48.29	47.86	48.04	0.408	0.906	0.855	0.932
MUFA^3^	39.32	39.57	40.42	39.50	0.400	0.472	0.564	0.464
PUFA^4^	12.89^*a*^	12.23^*ab*^	11.79^*b*^	12.44^*ab*^	0.295	0.081	0.205	0.040
ΣPUFA:SFA	0.27^*a*^	0.25^*ab*^	0.25^*b*^	0.26^*ab*^	0.047	0.175	0.250	0.085
Σn6 PUFA^5^	11.45^*a*^	10.79^*ab*^	10.51^*b*^	11.17^*ab*^	0.288	0.107	0.404	0.049
Σn3 PUFA^6^	0.52^*ab*^	0.56^*a*^	0.46^*b*^	0.46^*b*^	0.087	0.023	0.022	0.061
Σn6:n3 PUFA	22.36^*ab*^	19.79^*b*^	22.98^*ab*^	24.47^*a*^	0.562	0.032	0.073	0.039

*^1^Duncan, linear, and quadratic effects for HMB inclusion levels.*

*^2^SFA = C10:0 + C12:0 + C14:0 + C16:0 + C17:0 + C18:0 + C20:0.*

*^3^MUFA = C16:1 + C18:1n9t + C18:1n9c + C20:1 + C22:1n9.*

*^4^PUFA = C18:2n6c + C18:3n3 + C20:2 + C20:3n6 + C20:4n6.*

*^5^n3 PUFA = C18:3n3.*

*^6^n6 PUFA = C18:2n6c + C20:3n6 + C20:4n6.*

*^*a,b*^Values (n = 8) within a row with different superscripts differ significantly (P < 0.05).*

The results concerning the fatty acid composition of ASA were presented in Table 2. No significant difference was observed in the contents of C18:2 n6c, C20:3 n6, and C20:4 n6, the sum of n6 PUFA, SFA, MUFA, and PUFA, the ratio of PUFA to SFA, and the ratio of n6 to n3 PUFA (*P* > 0.05). HMB linearly increased the concentration of C18:3 n3, with the highest value observed in the 0.13% HMB group and the lowest value in 0.64% HMB group (*P* < 0.05).

**TABLE 2:** Fatty acid composition of abdominal subcutaneous adipose tissue in Bama Xiang mini-pigs fed the diets with various levels of HMB (% of total fatty acids).

Items	Dietary levels of HMB, %				SEM	*P*-value^1^		
	0	0.13	0.64	1.28		ANOVA	Linear	Quadratic
C10:0	0.13	0.11	0.12	0.14	0.057	0.310	0.591	0.057
C12:0	0.15	0.12	0.14	0.13	0.055	0.163	0.920	0.055
C14:0	1.88^*a*^	1.53^*b*^	1.73^*ab*^	1.86^*a*^	0.168	0.047	0.800	0.168
C16:0	27.92^*ab*^	26.76^*b*^	27.51^*ab*^	28.53^*a*^	0.352	0.038	0.222	0.352
C16:1	1.49	1.28	1.45	1.48	0.189	0.550	0.806	0.189
C17:0	0.24^*a*^	0.23^*ab*^	0.20^*b*^	0.20^*b*^	0.060	0.042	0.007	0.060
C18:0	19.81	20.77	20.77	19.97	0.462	0.661	0.874	0.462
C18:1 n9t	0.11	0.12	0.11	0.10	0.042	0.195	0.342	0.042
C18:1 n9c	34.80	36.00	35.61	35.30	0.427	0.550	0.679	0.427
C18:2 n6c	9.93	9.79	9.19	8.92	0.331	0.179	0.028	0.331
C20:0	0.31^*ab*^	0.28^*b*^	0.33^*a*^	0.28^*b*^	0.064	0.056	0.444	0.064
C20:1	1.61	1.44	1.57	1.54	0.219	0.895	0.888	0.219
C18:3 n3	0.49^*ab*^	0.50^*a*^	0.42^*b*^	0.43^*ab*^	0.086	0.062	0.022	0.086
C20:2	0.72	0.66	0.66	0.62	0.120	0.541	0.162	0.120
C20:3 n6	0.13	0.11	0.13	0.11	0.044	0.067	0.170	0.044
C22:1 n9	0.15	0.14	0.14	0.13	0.054	0.672	0.212	0.054
C20:4 n6	0.25	0.25	0.26	0.23	0.069	0.552	0.343	0.069
SFA^2^	50.44	49.79	50.80	51.11	0.479	0.643	0.369	0.479
MUFA^3^	38.15	38.98	38.88	38.55	0.453	0.815	0.716	0.453
PUFA^4^	11.52	11.30	10.65	10.31	0.350	0.149	0.020	0.350
ΣPUFA:SFA	0.23	0.23	0.21	0.20	0.056	0.189	0.034	0.056
Σn6 PUFA^5^	10.32	10.15	9.57	9.26	0.336	0.185	0.028	0.336
Σn3 PUFA^6^	0.49^*ab*^	0.50^*a*^	0.42^*b*^	0.43^*ab*^	0.086	0.062	0.022	0.086
Σn6:n3 PUFA	21.14	20.77	22.90	21.83	0.453	0.156	0.195	0.453

*^1^Duncan, linear, and quadratic effects for HMB inclusion levels.*

*^2^SFA = C10:0 + C12:0 + C14:0 + C16:0 + C17:0 + C18:0 + C20:0.*

*^3^MUFA = C16:1 + C18:1n9t + C18:1n9c + C20:1 + C22:1n9.*

*^4^PUFA = C18:2n6c + C18:3n3 + C20:2 + C20:3n6 + C20:4n6.*

*^5^n3 PUFA = C18:3n3.*

*^6^n6 PUFA = C18:2n6c + C20:3n6 + C20:4n6.*

*^*a,b*^Values (n = 8) within a row with different superscripts differ significantly (P < 0.05).*

### Colonic SCFA Concentrations

**FIGURE 3:**
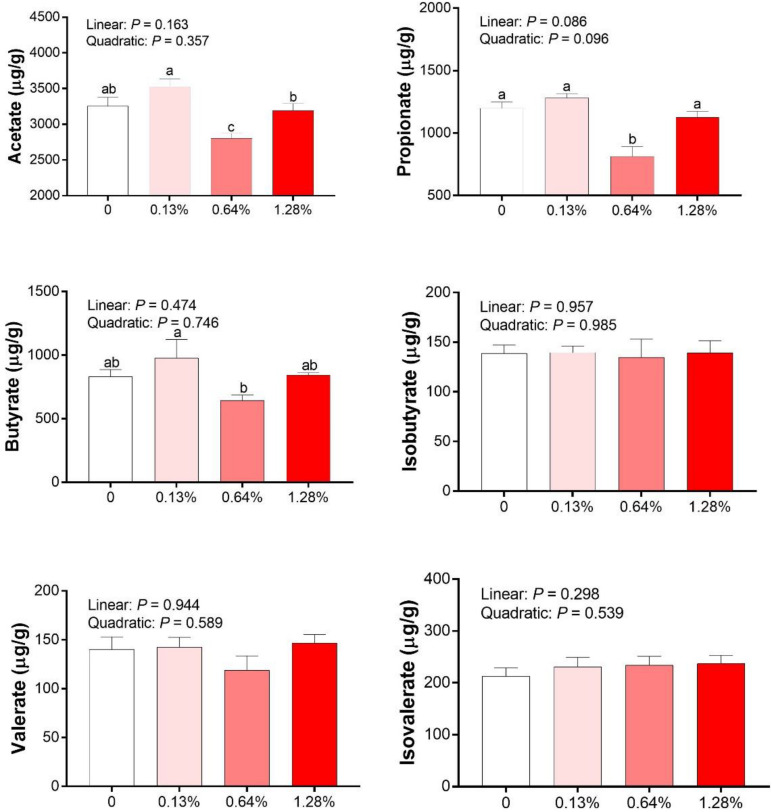
The SCFAs concentrations in the colonic contents of Bama Xiang mini-pigs. Data are presented as mean ± SEM (*n* = 8). ^*a,b,c*^ Values with different letters are significantly different among dietary HMB treatments (*P* < 0.05).

**FIGURE 4:**
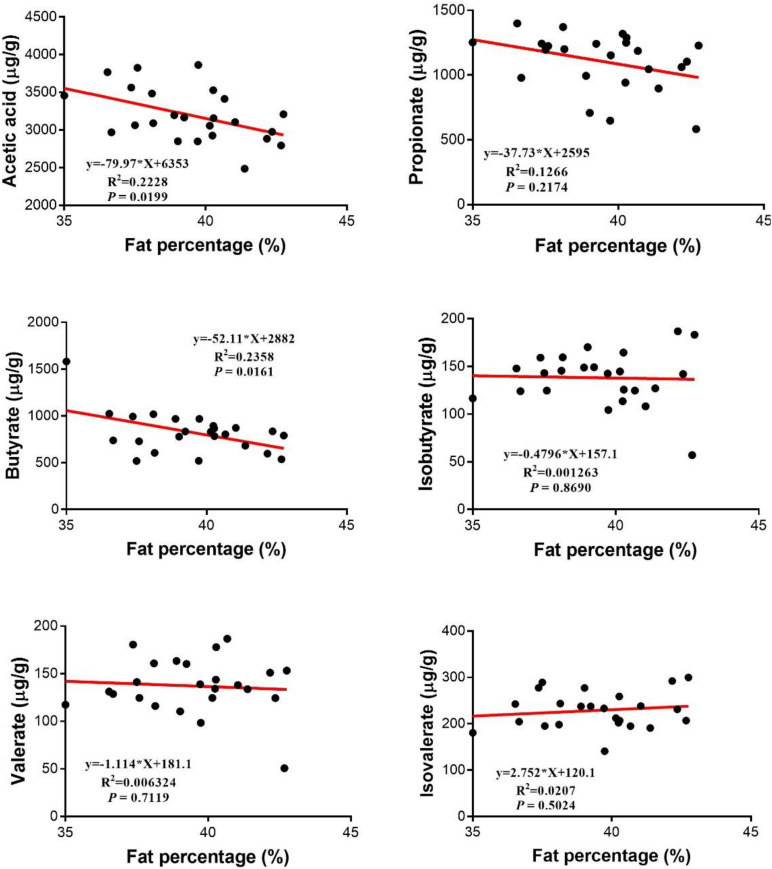
Relationship between SCFAs and the fat percentage. Pearson correlations were used to determine the association between the fat percentage and colonic acetate, propionate, butyrate, valerate, isobutyrate, and isobutyrate concentrations.

As shown in Figure 3, there were no linear or quadratic effects of increased HMB levels on colonic isobutyrate, valerate, and isovalerate concentrations (*P* > 0.05). The highest and the lowest concentrations of acetate, propionate and butyrate appeared in the 0.13% HMB group and 0.64% HMB group, respectively (*P* < 0.05). In addition, correlation analyses between the fat percentage and colonic acetate, propionate, butyrate, isobutyrate, valerate, and isovalerate concentrations were conducted by Pearson correlation analysis (Figure 4). The fat percentage negatively correlated with the colonic acetic acid and butyrate production (*P* < 0.05).

### Gut Microbiota Composition of Bama Xiang Mini-Pigs

**FIGURE 5:**
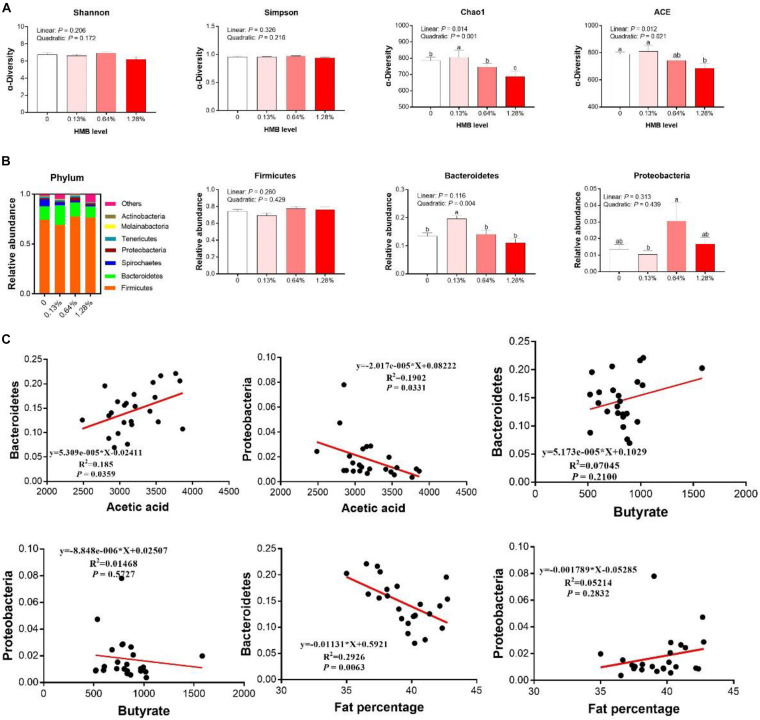
Dietary HMB supplementation improved gut microbiota in Bama Xiang mini-pigs. The α-diversity analysis **(A)**, microbiota composition at the phylum level **(B)**, the correlation analyses between the relative abundance of *Bacteroidetes*/*Proteobacteria* and acetic acid/butyrate, the correlation analyses between the relative abundance of *Bacteroidetes*/*Proteobacteria* and fat percentage **(C)**. Data are presented as mean ± SEM (*n* = 8). ^*a,b,c*^ Values with different letters are significantly different among dietary HMB treatments (*P* < 0.05).

As shown in Figure 5A, there were no significant differences in indexes of Shannon and Simpson among the groups (*P* > 0.05). However, linear and quadratic effects of HMB supplementation on indexes of Chao1 and ACE (Chao1, linear, *P* = 0.014, quadratic, *P* = 0.001; ACE, linear, *P* = 0.012, quadratic, *P* = 0.021) were observed, with the highest value observed in the 0.13% HMB group and the lowest value observed in the 1.28% HMB group. To further explore the overall microbial composition, we analyzed the relative abundance of dominant taxa at the phylum level. As shown in Figure 5B, the most dominant phyla in the bacterial communities were *Firmicutes*, *Bacteroidetes*, and *Proteobacteria*. HMB supplementation did not significantly affect the relative abundance of *Firmicutes* (*P* > 0.05). The relative abundance of *Bacteroidetes* reached its greatest value in the 0.13% HMB group and was higher than that in other three groups (quadratic, *P* < 0.05). The relative abundance of *Proteobacteria* was the highest in the 0.64% HMB group and the lowest in the 0.13% HMB group (*P* < 0.05). Furthermore, correlation analyses between the fat percentage and the relative abundances of *Bacteroidetes* and *Proteobacteria* were conducted by Pearson correlation analysis (Figure 5C). The acetic acid concentration was positively/negatively correlated with the abundance of *Bacteroidetes/Proteobacteria*, respectively (*P* < 0.05). Moreover, the abundance of *Bacteroidetes* was negatively correlated with the fat percentage (*P* < 0.05). No correlation was observed between the abundance of *Proteobacteria* and the fat percentage, and between the butyrate production and the relative abundance of *Bacteroidetes* or *Proteobacteria* (*P* > 0.05).

### Protein Expression of p-AMPKα, p-FoxO1, p-mTOR, and p-Sirt1

**FIGURE 6:**
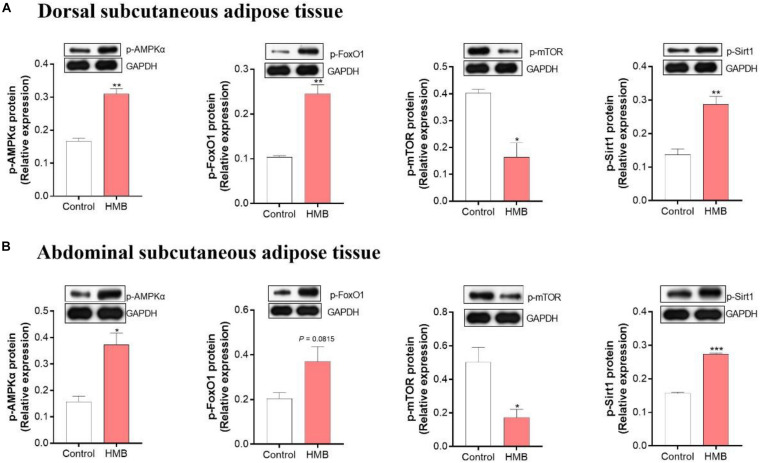
The expression of phosphorylation (p)-AMPKα, FoxO1, mTOR and Sirt1 in DSA **(A)** and ASA **(B)** of Bama Xiang mini-pigs fed diets with 0.13% HMB-Ca. Data are presented as mean ± SEM (*n* = 8). *P < 0.05, **P < 0.01, ***P < 0.001. AMPK, AMP-activated protein kinase; FoxO1, forkhead box O1; mTOR, mammalian target of rapamycin; Sirt1, silent information regulator 2 related enzyme 1.

The protein expression of p-AMPKα, p-FoxO1, p-mTOR, and p-Sirt1 in the control and 0.13% HMB groups were shown in Figure 6. In the DSA (Figure 6A), compared to the control group, the 0.13% HMB supplementation significantly upregulated the protein expression of p-AMPKα, p-FoxO1, and p-Sirt1 (*P* < 0.05), and downregulated the p-mTOR protein expression (*P* < 0.05). In the ASA (Figure 6B), compared to the control group, the 0.13% HMB supplementation significantly upregulated the protein expression of p-AMPKα and p-Sirt1 (*P* < 0.05), and downregulated the p-mTOR protein expression (*P* < 0.05). The protein expression of p-FoxO1 showed a trend to increase in response to HMB supplementation (*P* = 0.0815).

## Discussion

In the current study, we presented evidence that dietary supplementation of HMB with optimal level (0.13%) significantly inhibited fat deposition in Bama Xiang mini-pigs, as evidenced by the decreased fat percentage and adipocyte mean area of DSA. Notably, these observations did not occur at higher levels of HMB. In good agreement with our results, evidence from a rodent model has demonstrated that the maximum reduction of fat mass and mean size of WAT occurred at the HMB level of 1% (wt/vol), with higher concentrations of HMB being ineffective (Duan et al., 2019). Similar observations were also obtained in protein metabolism, with higher doses of HMB being ineffective (Wheatley et al., 2014). However, the reason why higher concentrations of HMB are unable to regulate lipid metabolism positively requires further interrogation. In addition, unlike DSA, HMB supplementation failed to reduce the adipocyte mean area of ASA. Therefore, these results suggest that the appropriate HMB dosage was 0.13% and that HMB mainly reduced the fat mass of DSA rather than of ASA.

Given the site differences in adipocyte size of subcutaneous fat depots following HMB supplementation, we speculated that there were differences in the fatty acid composition of subcutaneous fat depots in different regions. Moreover, there is compelling evidence that adipose tissue fatty acid composition is greatly associated with fat cell size (Raclot et al., 1997; Garaulet et al., 2006). It has been reported that n3 and n6 PUFAs are negatively correlated with the adipocyte size. In contrast, SFA is positively correlated with the adipocyte size (Garaulet et al., 2006). In the current study, although the SFA in DSA and ASA upon the HMB diets did not achieve the statistical significance, dietary HMB supplementation greatly elevated the n3 PUFA in selected WAT compared to the control group, and the maximum elevation of the n3 PUFA occurred at the HMB level of 0.13%. Unlike the n3 PUFA, HMB supplementation quadratically decreased n6 PUFA concentration in DSA, with the lowest value observed in the 0.64% HMB group, and did not significantly affect this parameter in ASA. These observations might underlie the mechanistic explanation for a beneficial role of HMB on the adipocyte mean area of DSA rather than ASA. Site differences in numerous metabolic activities in subcutaneous fat depots have been reported by studies in obese and non-obese humans (Lithell and Boberg, 1978; Malcom et al., 1989). Therefore, the differences in fatty acid composition between DSA and ASA indicate that HMB might differently affect metabolic activities (such as rate of deposition and mobilization) in the two subcutaneous sites.

It is well-known that the occurrence and development of obesity is closely associated with alterations in composition, diversity, and function of the gut microbiota (Shen et al., 2013; Gérard, 2015; ‘Graham et al., 2015). To explore whether dietary HMB supplementation reduced the fat deposition via gut microbiota, we analyzed the composition of gut microbiota in the colon of Bama Xiang mini-pigs. Our data showed that HMB supplementation (0.13%) increased the relative abundance of *Bacteroidetes*, and the increased *Bacteroidetes* was positively associated with the reduction of fat percentage. These results are well-matched with works in a rodent model (Duan et al., 2019). Moreover, our data showed that the relative abundance of *Proteobacteria* was the highest in the 0.64% HMB group and the lowest in the 0.13% HMB group. It has been reported that *Proteobacteria* is related to intestinal inflammation and gastrointestinal diseases (Kang et al., 2019; Bakhti et al., 2020; Zeng et al., 2020). Therefore, evidence from the current study shows that HMB supplementation at the level of 0.13% leads to the elevation of the beneficial bacteria *Bacteroidetes* and the reduction of the harmful bacteria *Proteobacteria*. Based on the results from our previous study (Duan et al., 2019) and the present study, we speculated that alterations in gut microbiota composition might contribute to the reduced fat deposition of Bama Xiang mini-pigs observed in the 0.13% HMB group. However, our knowledge of how HMB targets gut microbiota to reduce fat deposition is far from complete so that it needs to be further explored.

Several recent studies have demonstrated that the bacterial metabolites SCFAs exert a role in the action of gut microbiota (Yin et al., 2018; Duan et al., 2019; Hu et al., 2019), which motivated us to analyze the concentrations of SCFAS in the colon of Bama Xiang mini-pigs. Our data showed that HMB supplementation (0.13%) tended to reverse the decrease of acetic acid, and acetic acid production was negatively correlated with the fat percentage. These results are in accordance with other studies, which have demonstrated that diets-elevated colonic production of acetic acid contributes to the reduction of body weight in high fat diets-induced obese mice (Yin et al., 2018). Upon further investigation, we found that the colonic acetic acid production and the relative *Bacteroidetes* abundance showed a positive correlation. A major route to form acetic acid from dietary carbohydrates is driven by the abundant *Bacteroidetes* (Miller, 1978). Therefore, these results partially indicated that HMB supplementation affects gut microbiota compositions (especially for *Bacteroidetes*), which further generated acetic acid to reduce fat deposition. Strong evidence for the role of acetic acid on obesity comes from several rodent studies (Yamashita et al., 2007, 2009; Beh et al., 2017). In contrast to the results observed in Bama Xiang mini-pigs, data from rodent studies showed that the beneficial effects of HMB on obesity was mediated by gut microbiota-propionic acid axis (Duan et al., 2019). The inconsistency of the two reports might be due to the differences in experimental models, the experimental approaches, and the varied doses used in each experiment. This discrepancy suggests that results from rodent studies are not readily translated to human models of obesity.

Interestingly, there is evidence showing that the activation of AMPKα in adipose tissue could be one possible contributing mechanism for the beneficial effects of acetic acid (Fu et al., 2018; Liu et al., 2019). The enzyme AMPKα exerts key roles in regulating energy homeostasis (Shen et al., 2013; Yamashita, 2016). Evidence from *in vivo* and *in vitro* studies suggests that AMPKα activation in adipose tissue could suppressing lipogenesis by impairing the mTOR signaling (Gaidhu et al., 2009; Wang Q. et al., 2018) and promoting the phosphorylation of FoxO1 (Greer et al., 2007; Chen et al., 2010). On the other hand, the phosphorylation of AMPKα could promote lipolysis by activating Sirt1 directly (Canto et al., 2009). In the current study, Western blot analysis revealed that HMB markedly upregulated the phosphorylation of AMPK, FoxO1, and Sirt1, and downregulated the phosphorylation of mTOR in selected WAT. These results are perfectly in line with a recent study which reported that HMB might regulate lipid metabolism via the AMPKα-mTOR and AMPKα-Sirt1 signaling pathways in the perirenal adipose tissue of Large white × Landrace pigs (Duan et al., 2018).

## Conclusion

**FIGURE 7:**
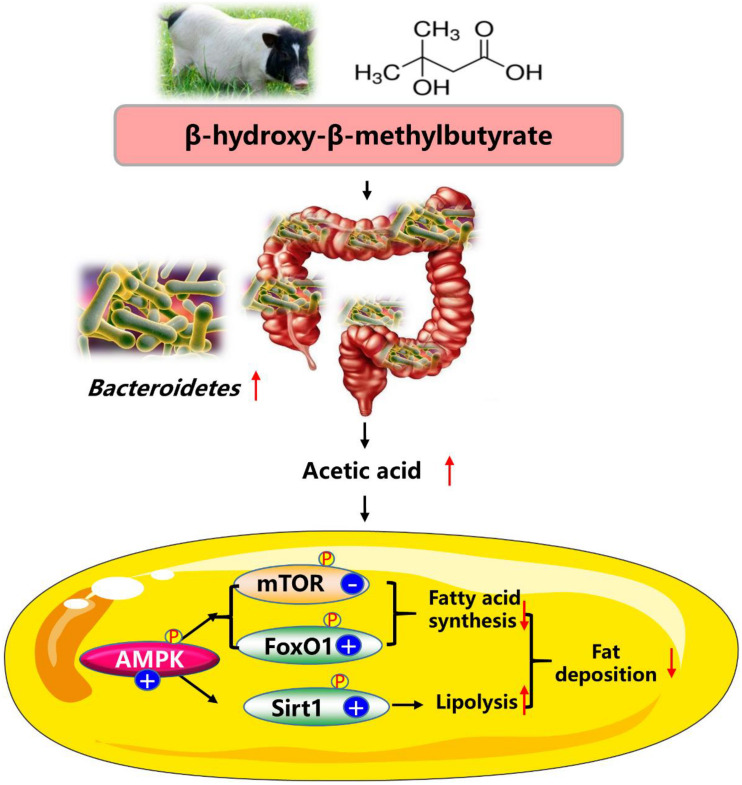
Proposed mechanisms of dietary HMB supplementation for modulating the lipid metabolism in adipose tissue of Bama Xiang mini-pigs. AMPK, AMP-activated protein kinase; FoxO1, forkhead box O1; mTOR, mammalian target of rapamycin; Sirt1, silent information regulator 2 related enzyme 1.

In summary, our data showed that dietary HMB supplementation at the level of 0.13% increased n3 PUFA and reduced fat mass in adipose tissue of Bama Xiang mini-pigs, and the favorable effects were more pronounced in the DSA. The beneficial effects of HMB on reducing fat deposition were likely regulated by the *Bacteroidetes*-acetic acid-AMPKα axis (Figure 7). These findings may provide valuable information for understanding the mechanisms of action of HMB in combating obesity. Areas that need further exploration include the research of mechanisms whereby HMB elevated the relative *Bacteroidetes* abundance and the acetic acid production. Although our data were encouraging, these findings should be confirmed with clinical studies with long-term follow-up.

## Data Availability Statement

The raw data supporting the conclusions of this article will be made available by the authors, without undue reservation.

## Ethics Statement

The animal study was reviewed and approved by Animal Welfare Committee of the Institute of Subtropical Agriculture, Chinese Academy of Sciences. Written informed consent was obtained from the owners for the participation of their animals in this study.

## Author Contributions

YD contributed to the conception of the study. JZ, CZ, BS, SZ, YZ, LZ, and GD performed the experiment. JZ and CZ contributed equally to data analysis and manuscript preparation. FL and QG revised the manuscript. All authors read and finalized the manuscript.

## Conflict of Interest

The authors declare that the research was conducted in the absence of any commercial or financial relationships that could be construed as a potential conflict of interest.

## Publisher’s Note

All claims expressed in this article are solely those of the authors and do not necessarily represent those of their affiliated organizations, or those of the publisher, the editors and the reviewers. Any product that may be evaluated in this article, or claim that may be made by its manufacturer, is not guaranteed or endorsed by the publisher.

## Abbreviations

AMPK α: AMP-activated protein kinase α
ASA: abdominal subcutaneous adipose tissue
DSA: dorsal subcutaneous adipose tissue
FoxO1: forkhead box O 1
HMB: β-hydroxy - β-methylbutyrate
mTOR: mammalian target of rapamycin
MUFA: monounsaturated fatty acid
PUFA: polyunsaturated fatty acid
SCFA: short chain fatty acid
SFA: saturated fatty acid
Sirt1: silent information regulator 2 related enzyme 1.
